# Numerical Modelling of Mixing in a Microfluidic Droplet Using a Two-Phase Moving Frame of Reference Approach

**DOI:** 10.3390/mi13050708

**Published:** 2022-04-30

**Authors:** Mesuli B. Mbanjwa, Kevin Harding, Irvy M. A. Gledhill

**Affiliations:** 1 Technology Transfer & Innovation Support, North-West University, Potchefstroom 2520, South Africa; mesuli.mbanjwa@nwu.ac.za; 2 School of Chemical and Metallurgical Engineering, University of the Witwatersrand, Johannesburg 2050, South Africa; kevin.harding@wits.ac.za; 3 School of Mechanical, Industrial & Aeronautical Engineering, University of the Witwatersrand, Johannesburg 2050, South Africa

**Keywords:** CFD, computational fluid dynamics, droplet-based microfluidics, droplet mixing, level set method, moving frame of reference, plug flow, slug flow, two-phase flow, simulation

## Abstract

Droplets generated in microfluidic channels are effective self-contained micromixers and micro-reactors for use in a multiplicity of chemical synthesis and bioanalytical applications. Droplet microfluidic systems have the ability to generate multitudes of droplets with well-defined reagent volumes and narrow size distributions, providing a means for the replication of mixing within each droplet and thus the scaling of processes. Numerical modelling using computational fluid dynamics (CFD) is a useful technique for analysing and understanding the internal mixing in microfluidic droplets. We present and demonstrate a CFD method for modelling and simulating mixing between two species within a droplet travelling in straight microchannel, using a two-phase moving frame of reference approach. Finite element and level set methods were utilised to solve the equations governing the coupled physics between two-phase flow and mass transport of the chemical species. This approach had not been previously demonstrated for the problem of mixing in droplet microfluidics and requires less computational resources compared to the conventional fixed frame of reference approach. The key conclusions of this work are: (1) a limitation of this method exists for flow conditions where the droplet mobility approaches unity, due to the moving wall boundary condition, which results in an untenable solution under those conditions; (2) the efficiency of the mixing declines as the length of the droplet or plug increases; (3) the initial orientation of the droplet influences the mixing and the transverse orientation provides better mixing performance than the axial orientation and; (4) the recirculation inside the droplet depends on the superficial velocity and the viscosity ratio.

## 1. Introduction

Droplet-based microfluidic systems have attracted significant attention as viable tools for performing a range of complex functions in chemistry, biology, and nanotechnology, such as bioanalysis [1], chemical synthesis [2,3], cytometry [4], enzyme immobilisation [5], and synthesis of specialised microparticles and nanoparticles [6]. Microfluidic droplets function as self-contained micromixers and micro-reactors with compelling functionalities, such as precise control of reagent volume, narrow size distribution, and replicability of processes within individual droplets [7]. Rapid and effective mixing within droplets is essential in order to achieve high throughput and increased production rates. Dimensional scaling and segmented flow are inherent characteristics that are advantageous for both mass transfer and mixing.

Passive mixing within moving microfluidic droplets involves the transport of chemical species through both molecular diffusion and convection [8,9,10]. Convective mixing is driven by flow hydrodynamics within and outside the droplet [11,12]. Droplets and plugs travelling in microchannels exhibit certain internal hydrodynamics and circulatory flow due to various degrees of confinement by the microchannel walls and the resulting shear effects [13,14]. The recirculating flow inside the droplets and plugs promotes good mixing; however, as the length of the plug increases, the mixing efficacy is impeded [15]. Fluid properties, such as fluid viscosity [16] and viscosity ratio [17] also influence the droplet mixing phenomena.

A higher degree of passive mixing in a droplet or plug can be achieved in a shorter channel length by introducing serpentine passages in which mixing is dependent on channel shape [18], micromixers [19], obstacles [20] or grooves, and chaotic mixing [21,22].

Previous experimental studies have investigated hydrodynamics and mixing within microfluidic droplets and plugs using various imaging and light-based techniques. These methods include particle image velocimetry (µPIV) [15], particle shadow velocimetry [23], time-averaged fluorescence [8], fluorescence lifetime imaging [24], the schlieren technique [25], and visualisation using the colour of chemical species [12]. These techniques require specialised and generally expensive equipment such as high-speed cameras, laser systems, and microscopes to undertake the experimental investigations.

Computational fluid dynamics (CFD) provides both an alternative and a complementary approach to experimental techniques for investigating hydrodynamics and mixing phenomena and microfluidic droplets. Mixing processes in droplet microfluidics often involve three-way coupled physics of two-phase flow and mass transport (convection and diffusion) of chemical species, governed by a set of partial differential equations, which require simultaneous solutions. Advances in computer sciences and computational capabilities have made it possible to use numerical models and simulations for investigating complex physics and biological phenomena, such as those undertaken in microfluidic systems, through in silico experiments [26,27,28]. There are a number of commendable reviews on numerical modelling and simulation of two-phase and multiphase flows in microchannels and their applications [29,30,31]. Furthermore, some reviews concerning modelling and simulation of heat transfer in two-phase flow in microchannels also provide valuable insight into the hydrodynamics aspects [32,33,34].

Significant work using CFD with the volume of fluid method includes that of Wang et al. [35], demonstrating that a high degree of mixing in a droplet or plug travelling in serpentine microchannel occurs in a shorter distance than in a straight microchannel.

Confinement can result in both 2D and 3D flow effects within the droplet. In the case of rectangular microchannels, the 3D flow effects are most prominent when the aspect ratio between the width and the channel is approximately equal to one, due to the x-velocity and the y-velocity experiencing little or no influence from the z-velocity [36]. Some studies found that the results of liquid–liquid two-phase flow in a square microchannel in 2D were comparable to the results in 3D [37]. Experimental studies using µPIV have demonstrated that a two-dimensional section can provide useful information on velocity distribution and mixing within droplets travelling in rectangular microchannels [38,39]. In the current work, we use a CFD model based on a moving frame of reference (MFR) to examine mixing behaviour in a plug travelling in a straight microchannel. In the MFR approach, the flow field is modelled in the frame of reference of the moving droplet [40,41].

A secondary aspect of the work is to test the use of the level set method (LSM) for this application. The LSM offers the advantage of modelling a sharp interface without adjusting the mesh. As a first step towards 3D models, a 2D model is explored in this paper.

## 2. Model Description

### 2.1. Two-Phase Moving Frame of Reference

**Figure 1:**
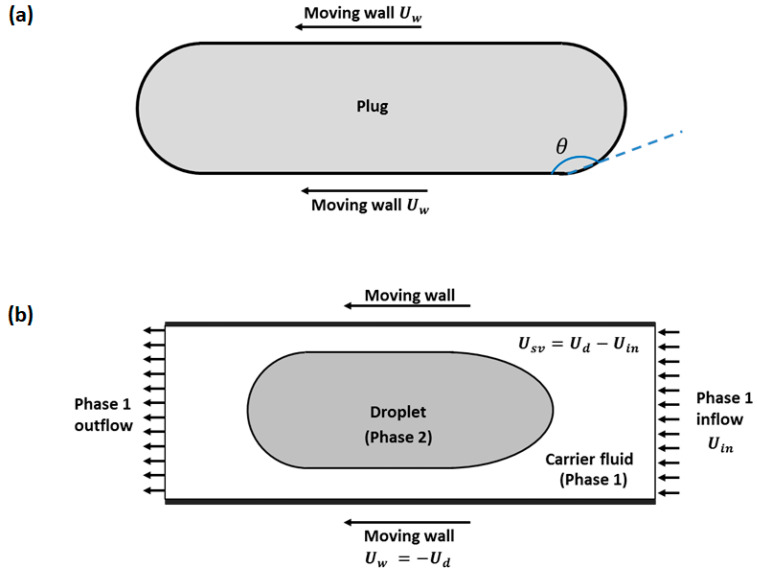
Computational domain for modelling droplet or plug transport in a two-phase fixed frame of reference. (**a**) Single-phase moving frame of reference and (**b**) two-phase moving frame of reference.

The approaches used in the modelling and simulation of two-phase flows are divided in two main categories, based on the computational domains used: (1) the fixed frame of reference and (2) the moving frame of reference. The fixed frame of reference is a Eulerian approach where the physics in the flow field are evaluated through a fixed window of observation. The moving frame of reference is a Lagrangian approach where the flow field is considered relative to the moving droplet. The moving frame of reference is also divided into two: (1) the single-phase moving frame of reference and (2) the two-phase moving frame of reference. Computationally, the single-phase moving frame of reference is the simplest and least costly of the three approaches. As illustrated in Figure 1a, the droplet or a plug is modelled as a single phase whose flow field is driven by a moving walls boundary condition. The model focusses on a single flow field either inside or outside the droplet or plug [36,42]. Consequently, fewer equations are solved than in a two-phase model, leading to faster results. However, the approach involves simplifying assumptions such as neglecting hydrodynamics in the second phase and the wetting film around the droplet [34]. Additionally, a priori assumptions are required regarding the shape of the interfaces and shear rates, typically based on existing experimental or numerical data. Where such data are not available, such foundational assumptions can be difficult or implausible to make. This method is also limited to cases where there is no wetting film between the plug and channel wall, or else it is disregarded.

The two-phase moving frame of reference (Figure 1b) is a compromise between the single-phase moving frame of reference and the fixed frame of reference. The two-phase moving frame of reference works on a computational domain smaller than the two-phase fixed frame of reference. It is also more versatile in application compared to the single-phase moving frame of reference, especially where capturing of interface, wall shear, and flow recirculation are important [40]. The considered domain captures the interfaces, a single plug or multiple plugs, and the surrounding flow fields, including the second phase.

### 2.2. Numerical Tools

The modelling of the two-phase flow was achieved using the LSM available in COMSOL Multiphysics software (version 3.5a), which is a versatile tool for handling coupled physics. In contrast to many commercial CFD programs which utilize finite volume discretization, COMSOL is based on the finite element method (FEM). COMSOL has been effectively utilised for CFD modelling in various microfluidic studies involving two-phase flow [43,44] and mixing [44,45]. The traditional LSM is a sharp interface (zero thickness), Eulerian method for modelling the evolution of an interface between two phases [46]. One of the main advantages of the LSM is its ability to handle topological changes and complex interfacial geometries, such as those seen during formation, deformation, and splitting of droplets. In LSM the interface is implicitly represented by the level set function (ϕ) and its evolution is governed by the level set (LS) transport equation [46]. A variant of the LSM, referred to as the conservative level set method, was proposed by Olsson and Kreiss [47,48]. The conservative LSM follows a similar approach to the colour function volume-of-fluid [31]. As with the general LSM, the evolution is of the interface is described by the level set equation. The LS function (ϕ), which represents the evolution of the interface, changes rapidly from 0 to 1 between the phases as a function of a smoothed Heaviside function. The interface has a nominal value of 0.5 [48]. The work on a validation test for the chosen numerical tools and approach has been reported elsewhere [49]

### 2.3. Governing Equations

#### Two-Phase Flow

The interface between fluids evolves due to fluid mechanics, and is governed by the level set transport equation, given by [46]:

(1) $$\frac{\partial \phi }{\partial t}+u\cdot \nabla \phi =0$$

where u=u(x,t) is the velocity field, x=(x,y,z) represents the Cartesian coordinate system, and t is the time variable. The interface is represented by the level set function ϕ=ϕ(x,t), and is initialised at a certain distance from the interface which satisfies a condition of the distance function |∇ϕ|=1. The level set equation for incompressible flow can be written in the following conservative form:

(2) $$\frac{\partial \phi }{\partial t}+\nabla \cdot \left(u\phi \right)=0$$

where ϕ is a function of a smoothed Heaviside step function ${ℋ}_{sm}\;\;$[48]:

(3) $$\phi ={ℋ}_{sm}\left(\phi_{sd}\right)=\{\begin{array}{ccc} 0, & & \phi_{sd}<-\varepsilon \;\\ & \frac{1}{2}+\frac{\phi_{sd}}{2\varepsilon }+\frac{1}{2\pi }\sin\left(\frac{\pi \phi_{sd}}{\varepsilon }\right), & -\varepsilon \leq \phi_{sd}\leq \varepsilon \\ 1, & & \phi_{sd}>\varepsilon \end{array}$$

where $\phi_{sd}$ is the signed distance function:

(4) $$\phi_{sd}\left(x\right)=\underset{x_{\Gamma }\in \Gamma }{\min}\left(\left|x-x_{\Gamma }\right|\right)$$

The ε parameter represents the interface thickness where ϕ smoothly transitions from 0 to 1, and is typically specified to be approximately equal to half the size of the mesh [50].

The velocity vector u is obtained from the solution of the flow equations for incompressible flow:

(5) $$\rho \left(\frac{\partial u}{\partial t}+u\cdot \nabla u\right)=-\nabla p+\nabla \cdot \mu \left[\nabla u+{\left(\nabla u\right)}^{T}\right]+\rho g+F_{\sigma }$$

(6) ∇u=0

where ρ and μ are the viscosity and density of the fluid, respectively; p=p(x,t) is the pressure, g is gravity and the superscript T denotes a matrix transpose function. In the current system, the effect of gravity is negligible and therefore the gravity term becomes zero.

The interfacial tension $F_{\sigma }$ is modelled using the continuum surface force (CSF) method [51]. Although physically the interfacial tension and curvature at the interface between the two fluids are boundary conditions, the CSF method permits them to be introduced in the Navier–Stokes equations via the body force term $F_{\sigma }$. The interface normal vector and interface curvature are given as [31]:

(7) $${\hat{n}}_{\Gamma }=\frac{\nabla \phi }{\left|\nabla \phi \right|}$$

(8) $$\kappa =-\nabla \cdot {\hat{n}}_{\Gamma }=-\nabla \cdot \left(\frac{\nabla \phi }{\left|\nabla \phi \right|}\right)$$

Therefore, $F_{\sigma }$ is expressed using a diffuse interface approach as:

(9) $$F_{\sigma }=\sigma \kappa \delta_{sm}{\hat{n}}_{\Gamma }$$

where $\delta_{sm}$ is a smoothed Dirac delta function, implemented in COMSOL Multiphysics™ as:

(10) $$\delta_{sm}\left(\phi \right)=6\left|\nabla \phi \right|\left|\phi \left(\phi -1\right)\right|$$

The density (ρ) and viscosity constants (μ) for the respective fluids are varied smoothly across the fluid–fluid interface according to:

(11) $$\rho =\rho_{2}+\left(\rho_{1}-\rho_{2}\right)\phi$$

(12) $$\mu =\mu_{2}+\left(\mu_{1}-\mu_{2}\right)\phi$$

The simplifying assumptions for the model were postulated as follows:

- The flow is Newtonian and isothermal.
- There are zero gradients for interfacial tension and hence, negligible Marangoni effects.
- The system is single droplet system (the flow field is a result of a single droplet).
- Viscosity and density are constant in each phase and are not functions of concentration of the species.
- Mass transport is confined within the droplet phase and is no interfacial mass transfer.

### 2.4. Mass Transport

In incompressible Newtonian flow, the density is constant and the velocity field is divergence free. When there are no effects contributing to the addition or consumption of the chemical species in the system, such chemical reaction or phase change, i.e., the source term is zero and thus the mass transport of chemical species is described by the passive scalar diffusion-convection equation [52]:

(13) $$\frac{\partial C_{i}}{\partial t}+u\cdot \nabla C_{i}-D_{i}\nabla^{2}C_{i}=0$$

where $C_{i}$ is the concentration of the transported chemical species i and $D_{i}$ is the mass diffusivity or diffusion coefficient of the transported chemical species i. In the above equation, the first term is the transient term (rate of change of concentration) which determines the net gain or loss of chemical species per unit time. The convective term determines the transport of chemical species by advection due to the velocity field **u.** The diffusive term describes the spatial dispersion or distribution of $C_{i}$, driven by the concentration gradient ($\nabla C_{i}$) and the diffusion coefficient. The transport equation is coupled to the flow (Navier–Stokes) equations via the velocity field.

### 2.5. Model Geometry

**Figure 2:**
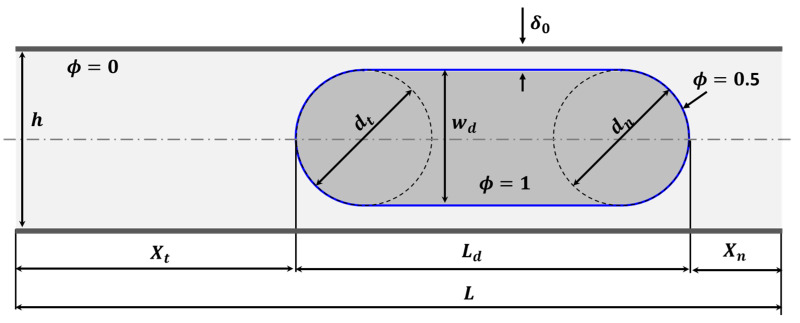
Schematic representation of the 2D model geometry for the current problem.

The model geometry for the problem is set up in 2D as shown in Figure 2. The droplet (plug) is positioned along the centre line of a microchannel with length L and height h. The droplet is specified as a composite geometric structure comprising a rectangle capped by two semi-circles of equal diameter at the front and back ends. The plug has a length of $L_{0}$ and a width of $w_{0}$. As such, the nose and the tail are of equal curvatures ($d_{n}=d_{t}$). The distance between the plug tail and the back edge of the microchannel is $X_{t}$ and $X_{n}$ is the distance between the nose the front end of the microchannel.

The channel height was fixed at h=100 μm while $L_{0}$ varied between 1.5h and 5h. The width of the droplet, as well as $d_{n}$ and $d_{t}$, were also constant at 0.8h. The thickness of the lubrication film is specified as $\delta_{0}=0.5\;\left(h-w_{0}\right)$. The microchannel length depended on $L_{0}$, $X_{t}$, and $X_{t}$, where $X_{t}=2h$, and $X_{n}=0.5h$.

### 2.6. Dimensionless Numbers

The capillary number and the Reynolds numbers are quantified in terms of the superficial velocity and the properties of continuous phase as $Ca=\mu_{c}U_{s}/\sigma$ and $Re=\rho_{c}U_{s}h/\mu_{c}$. The material properties of the continuous and the dispersed phases are compared through the viscosity ratio ($\lambda =\mu_{d}/\mu_{c}$) and the density ratio ($\psi =\rho_{d}/\rho_{c}$). The thickness of the film ($\delta_{f}$) is scaled by h such that $\delta^{*}=2\delta_{f}/h$ is the non-dimensionalised film thickness.

### 2.7. Numerical Implementation

#### 2.7.1. Computation of the Flow Field

**Figure 3:**
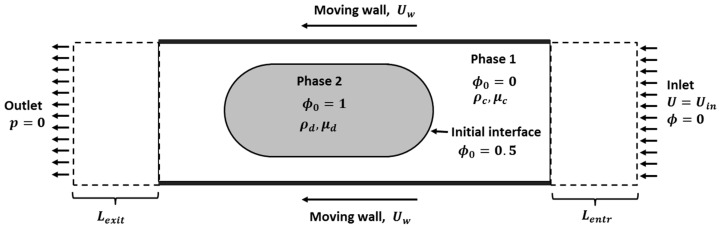
Initial and boundary conditions for a droplet travelling in microchannel modelled using a two-phase moving frame of reference.

A triangular mesh with a maximum size of $10^{-6}\;m$ was specified in the domain. The mesh at the walls was specified to have a maximum size of $2\times 10^{-7}\;m$. The specified mesh was selected following mesh independence tests for both the flow and the convection-diffusion solutions. The flow field and the concentration field were computed sequentially, with the flow solution being computed first. Initial and boundary conditions for the flow solutions were imposed, as illustrated in Figure 3.

The superficial velocity of the system is defined by the difference between the droplet velocity and the average inlet velocity, i.e., $U_{s}=U_{d}-U_{in}$. The speed of the dispersed phase relative to the superficial velocity is given by the droplet mobility $ℳ=U_{d}/U_{s}$. When ℳ=1, the droplet and the carrier travel with the same speed, typically when there is no lubrication film and the plug is fully wetting to the walls of microchannel [53]. The current problem considers the case where ℳ>1 and a lubrication film exists between the droplet and the channel wall. Necessarily, for the current problem the ℳ>1 condition must always prevail due to the moving wall boundary conditions. The effects of gravity and Marangoni effects were neglected. A density of 1000 kg/m^3^ and an interfacial tension of 0.014 N/m were specified.

The capillary numbers varied between 0.003 and 0.21, in proportion to the superficial velocity, which was adjusted by changing the wall velocity. Under constant interfacial tension conditions, the capillary number changes with changes in viscosity and in superficial velocity. The effect of different droplet size on mixing was investigated by setting the initial size of the droplet ($L_{d}$) to h, 2h and 5h. The viscosity ratio of 0.5 was used for the droplet size study. The effect of the viscosity ratio was investigated at single initial droplet size of $L_{0}=2h$. The viscosity of the continuous phase was kept constant for all of the simulations, at 0.002 Pa∙s. The computations for both the two-phase flow and convection–diffusion equations were performed using the parallel direct solver (PARDISO). The time steps for all cases were set to conform to a CFL condition of 0.25.

**Figure 4:**
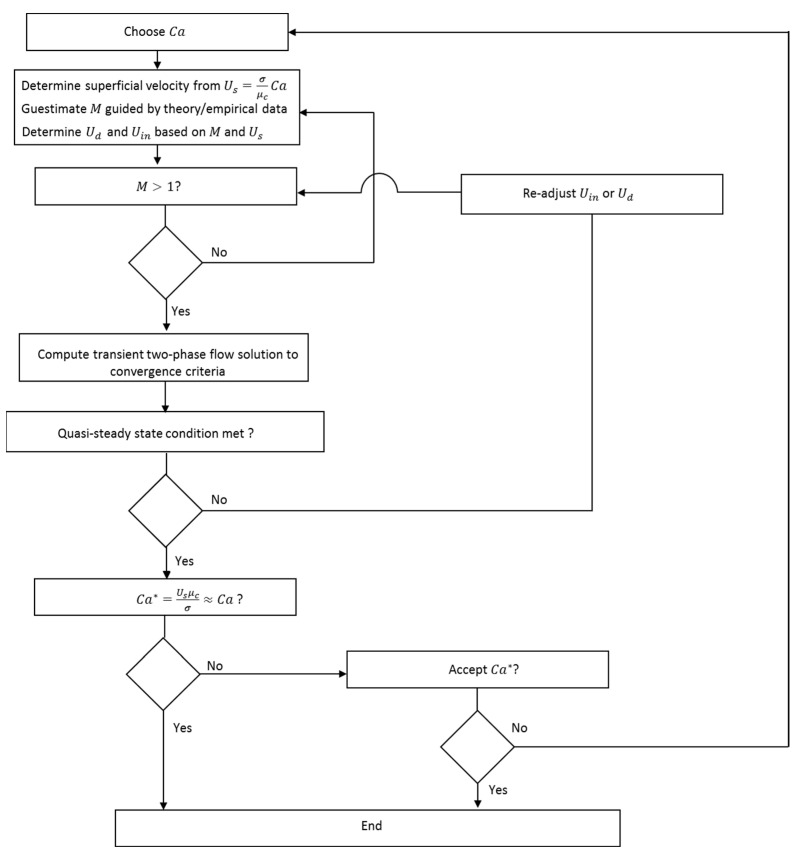
Flow diagram for the iteration process towards quasi-steady-state of the flow solution.

**Figure 5:**
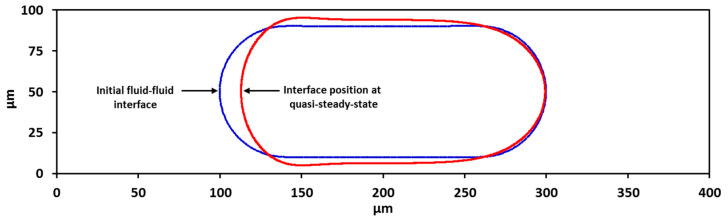
Position of fluid–fluid interface at quasi-steady-state versus its position at initial condition.

The values of $U_{s}$, $U_{d}$, and $U_{in}$ were determined iteratively from Ca and an initial estimate of droplet mobility and the solution was computed until a quasi-steady-state condition was achieved. The true steady state or equilibrium for the simulation prevails when the displacement of the nose of the droplet in the axial direction is zero [41]. The condition set for the quasi-steady-state is $\left|U_{drift}\right|/U_{sv}\leq 10^{-2}$ with a minimum of 1000 time steps, where $U_{drift}$ is the speed at which the nose moves at a given $U_{w}$ and $U_{in}$. The prevailing Ca values were then determined from the quasi-steady-state $U_{s}$. The flow diagram in Figure 4 shows the iterative process towards achieving the desired quasi-steady-state condition. Figure 5 illustrates the position of fluid–fluid interface at quasi-steady-state versus its position at initial condition.

#### 2.7.2. Computation of the Concentration Field

The concentration field was computed transiently by solving the convection–diffusion equation, coupled to the velocity field to flow solution. The velocity field for the computation of the concentration field was obtained from the quasi-steady-state solution of the two-phase flow. The computation of the concentration field C(x,y, t) was confined solely to the droplet phase (ϕ>0.5) by imposing subdomain and initial conditions which are appropriate for the model. The convection of the solute species into the carrier phase was minimised by making the x and y velocity components three orders of magnitude smaller than in the droplet phase, i.e.,

(14) $$u_{x}={u_{x}|}_{\phi >0.5}+\left({u_{x}|}_{\phi \leq 0.5}\right)\times 10^{-3}$$

and

(15) $$u_{y}={u_{y}|}_{\phi >0.5}+\left({u_{y}|}_{\phi \leq 0.5}\right)\times 10^{-3}$$

where $u_{y}$ and $u_{y}$ are x and y components of the velocity field. The second terms in the expressions minimise numerical errors which emanate from large concentration and velocity discontinuities the interface. Instead of the velocity components being set zero in the continuous phase, they are limited to three orders of magnitude smaller than the components in the droplet phase. The diffusion of solute species across the interface is constrained by imposing variable diffusion coefficients between the two phases:

(16) $${D|}_{\phi >0.5}=D_{0}$$

and

(17) $${D|}_{\phi \leq 0.5}=D_{0}\times 10^{-2}$$

**Figure 6:**
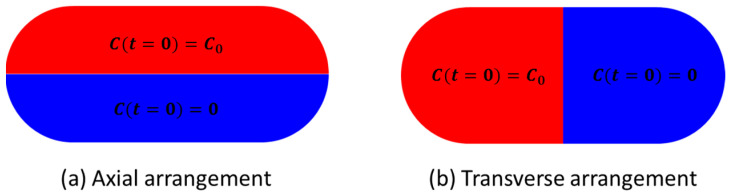
Flow diagram for the iteration process towards quasi-steady-state of the flow solution. (**a**) Axial arrangement; (**b**) Transverse arrangement.

For the current study, $D_{0}=10^{-9}\;m^{2}/s$. As with the case on velocity components, the second term accounts for the diffusion coefficient in the carrier phase which is very small but not equal to zero. The initial distribution of concentration of the solute species is oriented either transversely or axially to the flow direction as illustrated in Figure 6.

The transverse orientation is preferable, as it provides more efficient mixing inside droplets and plugs than the axial orientation [11,36]. In order to achieve transverse orientation, some microfluidic operations involve merging two droplets consisting of the fluid elements to be mixed in a merged droplet.

The influence on mixing by a number of parameters was investigated through a parametric study. The effect of the density ratio was excluded from the study and therefore kept constant at unity for all cases unless specified otherwise. Results from numerical studies suggest that two-phase flows in microchannels at low Re and λ are independent of density ratio [53]. The Reynolds number, the dependent variable, was influenced and varied with the controlling parameters. These selected parameters were typical of operating conditions in microfluidic systems. The diffusion coefficient was isotropic and a value of $D=10^{-9}$ m^2^/s, typical of macromolecules and proteins, was used for all of the simulations.

## 3. Results

### 3.1. Droplet Mobility

**Figure 7:**
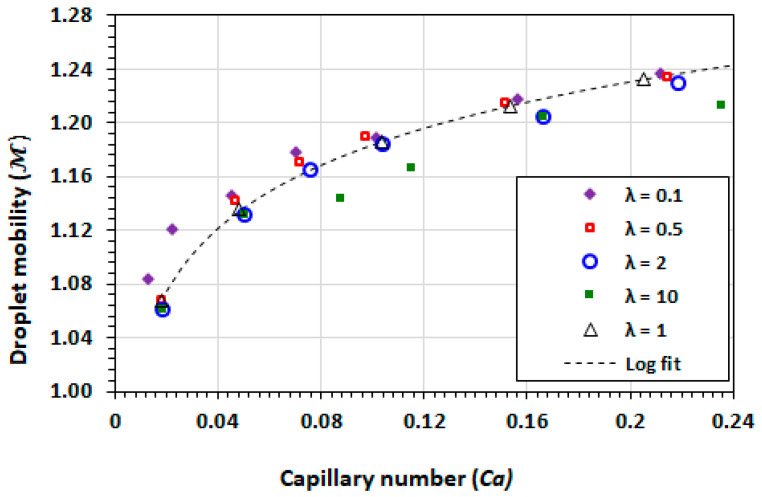
Plot of droplet mobility versus capillary number for different viscosity ratios.

Figure 7 shows the dependency relationship between the droplet mobility and capillary number at viscosity ratios of 0.1, 0.5, 1, 2, and 10. The droplet mobility was computed from the iteratively-determined flow parameters at a quasi-steady-state. For all the viscosity ratios the droplet mobility increases monotonically with an increasing capillary number. It is expected that for larger capillary numbers, the mobility would tend towards an asymptotic value upon which the data would diverge from the current curve fit. The significance of the results is that the parameters of the flow $U_{d}$, $U_{s},$ and ℳ can be stated a priori for other simulation studies within the *Ca* range.

The numerical data for the viscosity ratios in the range of 0.017<Ca<0.25 can be approximated by the curve ℳ=0.0675lnCa+1.339. The range of the mobility ratios are within the recirculating flow regimes as described in literature [54]. The lowest viscosity droplet (λ=0.1) generally has high mobility than other viscosity ratios at the same capillary number. At 0.02<Ca<0.13, the mobility values are between 1% and 3% higher than other viscosity ratios.

### 3.2. Droplet Shape and Size

**Figure 8:**
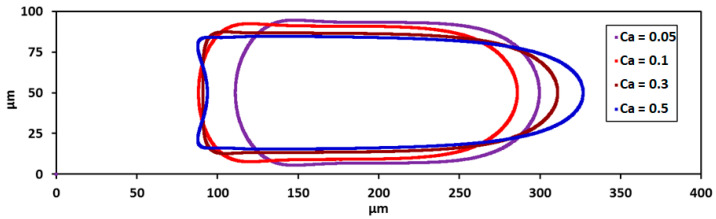
Shape of the interface of the droplet at various Ca values.

The shape of the plug is influenced by the dominance of the viscous force over the capillary forces. As the capillary number increases, the interfacial tension is not enough to retain large curvatures of the interface, the nose sharpens and the tail flattens (Figure 8). The tail end eventually curves inwardly as Ca increases to 0.5. The behaviour can also be attributed to increasing effect of inertial forces as the corresponding Re values are in the range of 6≤Re≤72 [55].

**Figure 9:**
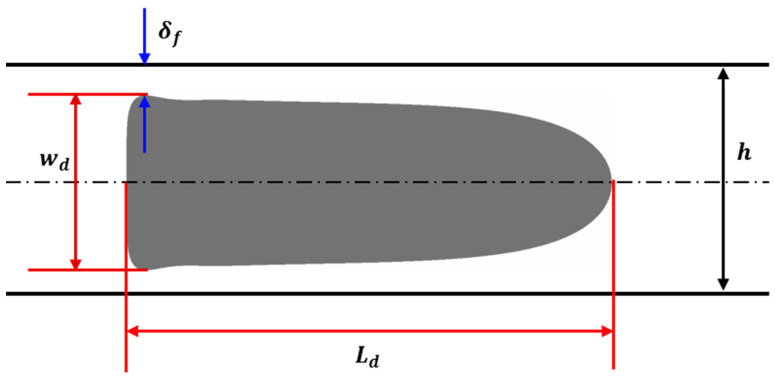
Measurement of the droplet length, width and minimum film thickness.

The size of the droplet is quantified via its length and width. The widest width dimension is used as the quantifying metric, given that the width of tapering droplets is not uniform. The thickness of the wetting film is based on the inverse of the same principle, i.e., minimum thickness. Figure 9 illustrates the measurement of droplet width, droplet length and minimum film thickness [53].

**Figure 10:**
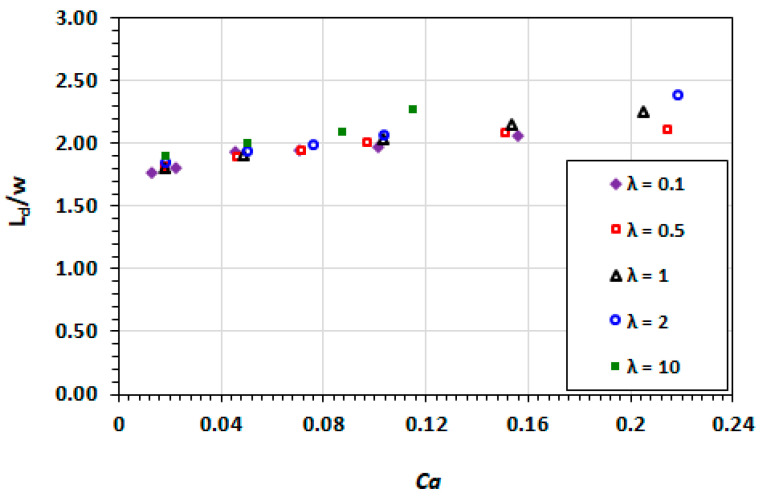
Dependence of the non-dimensionalised droplet length on capillary number at varying viscosity ratios.

The dependence of the non-dimensionalised droplet length on the capillary number is shown in Figure 10. The droplets become increasingly elongated as the capillary number increases. The droplets at viscosity ratios of 2 and 10 experience more deformation than the cases where λ≤1.

**Figure 11:**
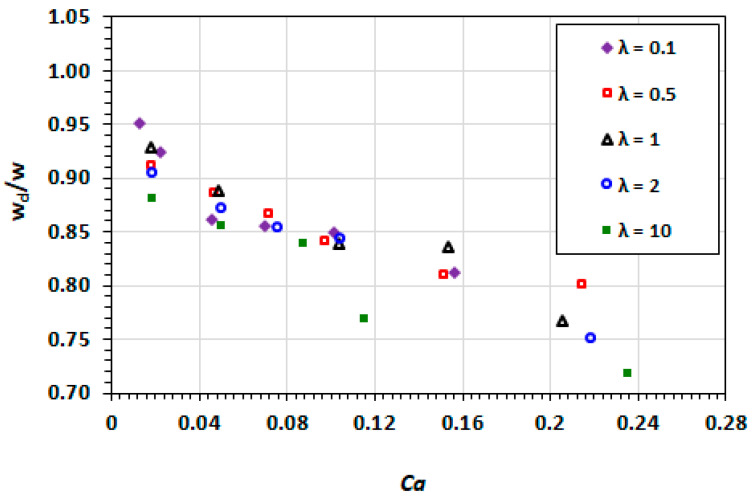
Dependence of the non-dimensionalised droplet width on capillary number at varying viscosity ratios.

Similarly, the width of the droplets decreases with an increasing capillary number, as shown in Figure 11. The contribution of the droplet viscosity in the overall domination of interfacial effects by viscous effects is demonstrated by the greater deformation of the droplets at λ>1.

**Figure 12:**
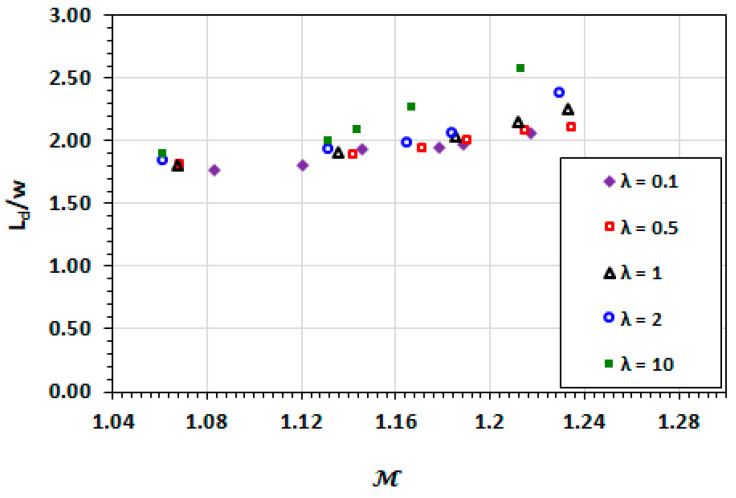
Dependence of the non-dimensionalised length on droplet mobility at varying viscosity ratios.

**Figure 13:**
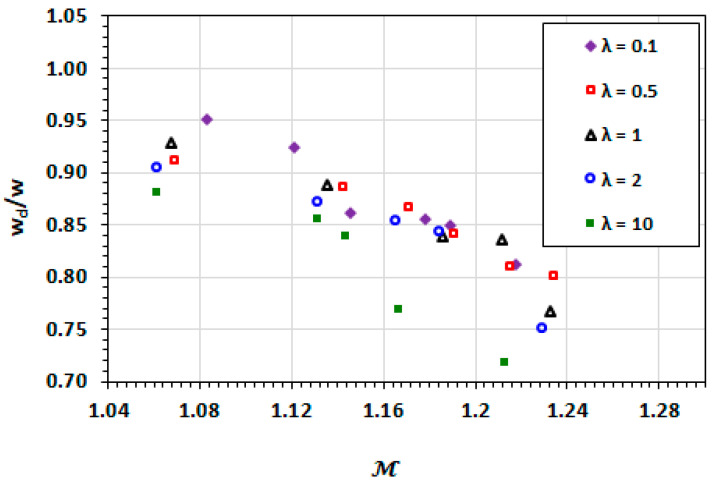
Dependence of the non-dimensionalised width on droplet mobility at varying viscosity ratios.

The dependencies of droplet length and width on droplet mobility are similar to those of the capillary number as shown in Figure 12 and Figure 13. The deformation (increasing length and narrowing width) of the droplet increases with the increasing droplet mobility. The high viscosity ratio exhibits greater deformation due to increased viscous effects. These viscous effects are not captured in the capillary number.

### 3.3. Film Thickness

**Figure 14:**
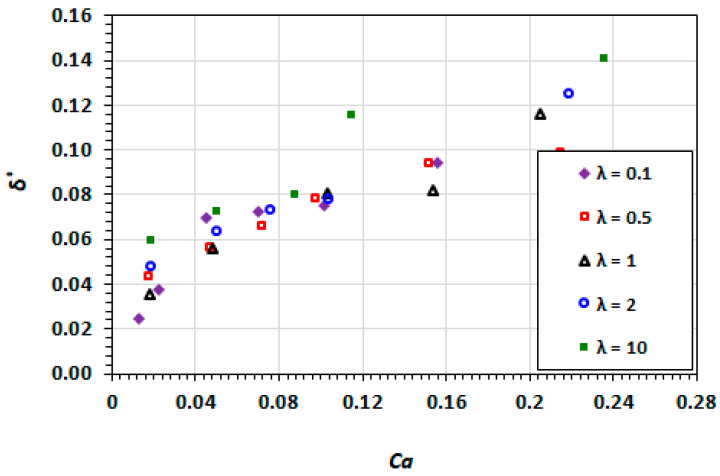
Dependence of the film thickness on capillary number at different viscosity ratios.

**Figure 15:**
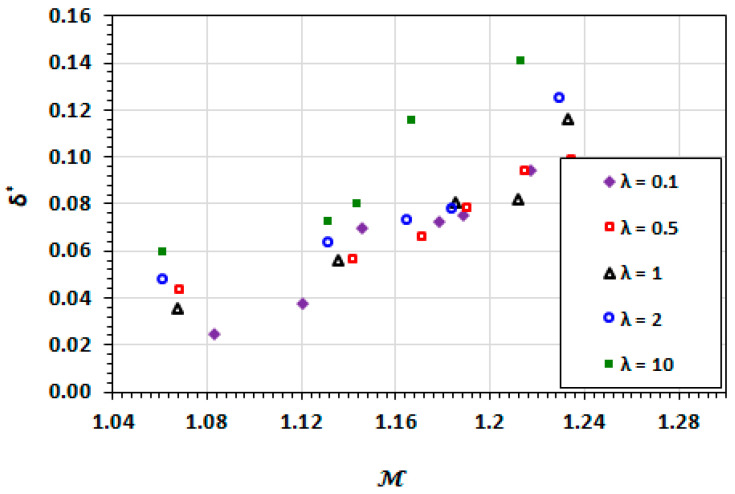
Dependence of the film thickness on droplet mobility number at different viscosity ratios.

Figure 14 and Figure 15 shows the results for the film thickness at various capillary numbers for a range of viscosity ratios. The minimum film thickness increases with an increasing capillary number. This is as a result of the viscous effects becoming more dominant the than the interfacial tension and therefore, the deformation of the interface. For Ca<0.1, the film thickness demonstrates minimal dependence on the viscosity ratio. However, there is a demonstrable influence of the viscosity of the droplet phase that is higher than the carrier phase, i.e., λ>1. The contribution of the viscosity of the droplet phase has traditionally been neglected in the analysis of the competition between the viscous and interfacial effects. The case in point is the determination of capillary number based only on the viscosity of the carrier phase. The current results suggest that in cases where the viscosity of the droplet phase is greater than unity, the capillary number may not be sufficient on its own to characterise the visco-capillary behaviour of the droplet flow.

Boudreaux [54] found that for capillary numbers above 0.3, the change in behaviour was present even for viscosity ratios of 0.5 and 1.

### 3.4. Velocity Field

**Figure 16:**
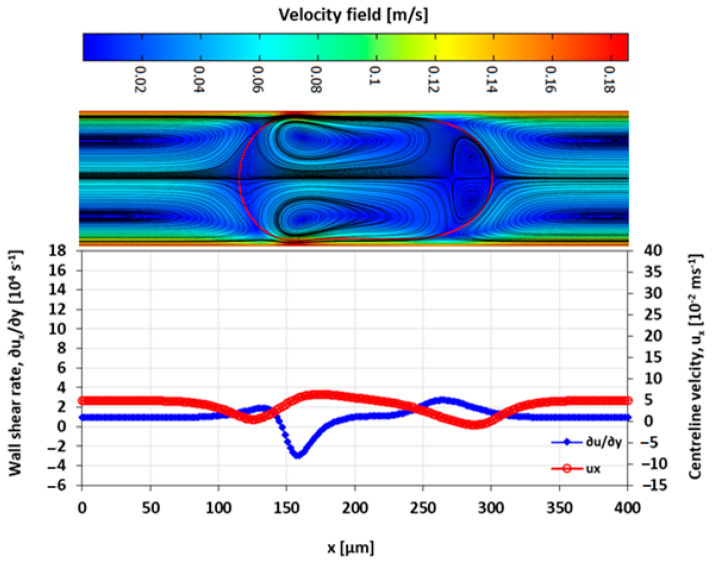
Wall shear rate and x-velocity along the centreline (y=h/2) at λ=2 and Ca=0.019.

**Figure 17:**
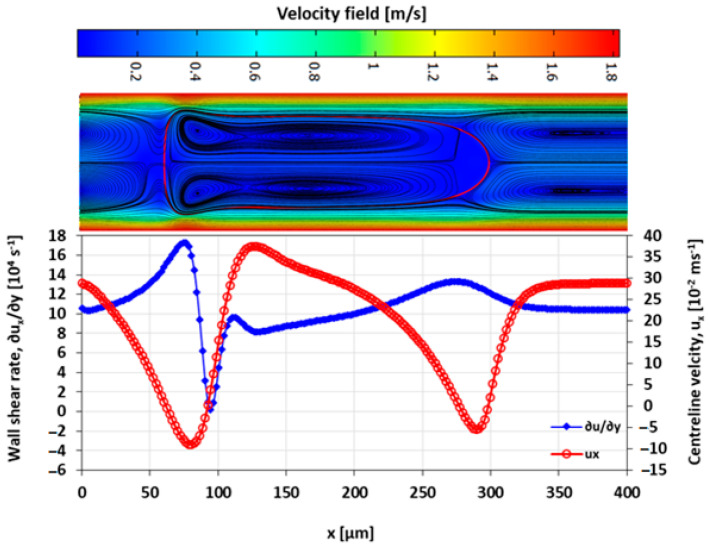
Wall shear rate and the x-velocity at centreline (y=h/2) for λ=2 and Ca=0.21.

**Figure 18:**
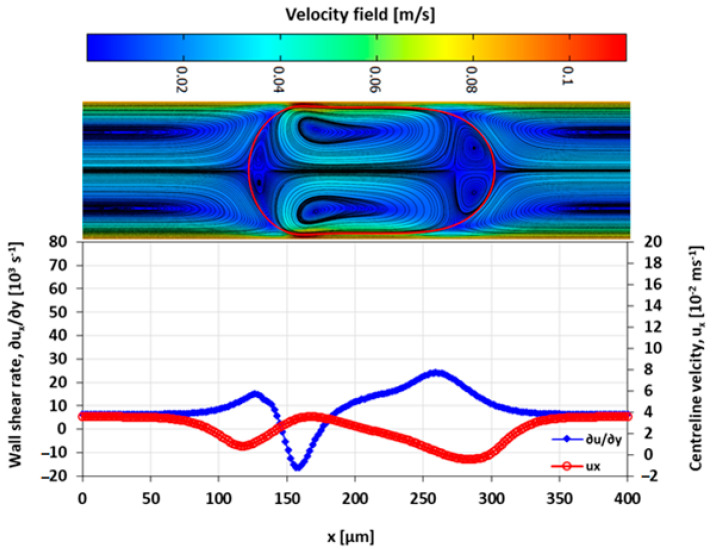
Wall shear rate and the x-velocity at centreline (y=h/2) for λ=0.5 and Ca=0.03.

**Figure 19:**
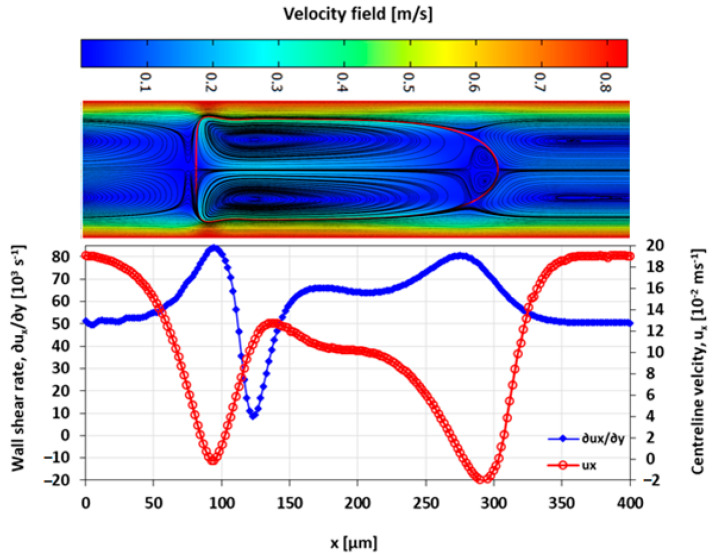
Wall shear rate and the x-velocity at centreline (y=h/2) for λ=0.5 and Ca=0.2.

The velocity field within the droplet largely drives the convective transport and mixing. The recirculating flow creates vortices symmetrical about the centre line along the *x*-axis. In diffusion dominated flow, the vortices enhance mixing by reducing the diffusion path length of the solute [10,42]. The recirculations lead to stagnation zones, where ∥u∥ → 0, which manifest as regions of poor mixing in convection-dominated mass transport. The viscosity ratio between the two liquid phases also influences the nature and the extent of the recirculation. The recirculating flow in the droplet is induced by the flow field of the carrier fluid. The source of the shear is the surrounding carrier fluid and the confining microchannel walls. The wall shear rate is approximately double for λ=2 than for λ=0.5 at the specified capillary numbers (Figure 16, Figure 17, Figure 18 and Figure 19).

The extent of the recirculation zone can be estimated from the intervals over which $u_{x}$ at the centreline drops below zero. At λ=2, the recirculation zone at the leading edge of the droplet remains approximately constant with Ca, but at λ=0.5 the cap length decreases with an increasing Ca, reducing the mixing performance.

### 3.5. Mixing Index

Generally, it is desirable for mixing operations to achieve the desired mixing in the quickest time and shortest channel distance. More often than not, the targeted result of mixing is to obtain a homogenous mixture (i.e., complete mixing) of the fluid elements or chemical species. The extent of mixing at various times has been quantified through the mixing index [56]:

(18) $$I_{M}=\left(1-\frac{\int_{\phi \geq 0.5}^{\;}\left|C-C_{\infty }\right|d\Omega }{\int_{\phi \geq 0.5\;}^{}\left|C_{0}-C_{\infty }\right|d\Omega }\right)$$

where dΩ is an area element of the 2D domain surface. The values of $I_{M}$ range from 0 to 1, where the maximum value indicates a complete mixing of the solute species to an average concentration of $C_{\infty }$ within the droplet. The average concentration is proportional to ratio of the area ($A_{0})$ covered by the initial maximum concentration to the total area of the droplet ($A_{\phi })$, such that:

(19) $$C_{\infty }=C_{0}\;\frac{A_{0}}{A_{\phi }}$$

In the current work $A_{\phi }$ and $A_{0}$ are determined by integration over the respective areas covered and therefore:

(20) $$C_{\infty }=C_{0}\left(\frac{\int_{\phi >0.5}^{}\int_{C_{0}}^{\;}d\Omega }{\int_{\phi >0.5}^{\;}\;d\Omega }\right)\;$$

This approach eliminates the need to have $C_{0}$ occupying precisely half of the droplet, and thus can be applied to cases where $A_{0}\neq 0.5A_{\phi }$.

### 3.6. Limitation of the Droplet Mixing Model

The problem at hand requires consideration of the mass transport within the droplet and not in the carrier phase. The manner in which this is achieved in the current method is that both the diffusion coefficient and the velocity field in the carrier phase are neglected. This achieved by imposing initial and subdomain conditions. There are some limitations on the model which relate to three key issues, namely: (1) loss of molar species (or mass loss) manifesting through unbalanced and unphysical decline in concentration, (2) droplet mixing cases where diffusion dominates over convective transport, and (3) leakage of chemical species from the droplet phase into the carrier phase. The origin of the first issue emanates from the well-known inherent disadvantage of the level set method being poor area (volume) conservation in the LSM [46]. In the current work, the conservative LSM used for two-phase flow has good conservation as demonstrated by the COMSOL implementation of the method by Olsson and Kreiss [47]. However, coupling of the convection–diffusion physics to the level set function from the two-phase flow solution and imposing of subdomain and initial conditions on the phases separated by ϕ=0.5 shows some conservation problems.

The second issue emanates from the discontinuities in mass transfer flux across the fluid-fluid interface (ϕ=0.5), as the result of the imposed subdomain and initial conditions. When diffusion becomes dominant over convective mass transport, the imposed condition on the velocity field, i.e,

(21) $$u_{x}=\;{u_{x}|}_{\phi >0.5}+\left({u_{x}|}_{\phi \leq 0.5}\right)\times 10^{-3}$$

and

(22) $$u_{y}=\;{u_{y}|}_{\phi >0.5}+\left({u_{y}|}_{\phi \leq 0.5}\right)\times 10^{-3}$$

The concentration field develops numerical instabilities at the interface. The instabilities manifest as spikes (C≫1) which can grow to $O10^{2}$ and above, leading to divergence of the numerical solution. Notably, in the case of pure diffusion, i.e.,

(23) $${u_{x}|}_{\phi \leq 0.5}=\;{u_{x}|}_{\phi >0.5}=0$$

and

(24) $${u_{y}|}_{\phi \leq 0.5}={u_{y}|}_{\phi >0.5}=0$$

The problem of instabilities does not arise and there is good mass conservation. The third issue arises as a result of the high concentration of chemical species at the interface for long periods. The high concentration gradient between fluid in the droplet and in the carrier phase drives the diffusion of traces of chemical species across the interface. This mass leakage is a function of time rather than of the mixing regime. The mass leakage is more prevalent in the diffusion-dominated case than in convection-dominated case as the mixing times are generally longer.

When conservation is examined for the convection-dominated mixing case =0.21, λ=0.1, it is observed that there is good conservation of the chemical species in the domain (after 6 ms, approximately 99% in the domain and 97% in the droplet). There is, however, some mass leakage from the droplet in the into the carrier phase, although fairly insignificant over the mixing period with 10 ms.

**Figure 20:**
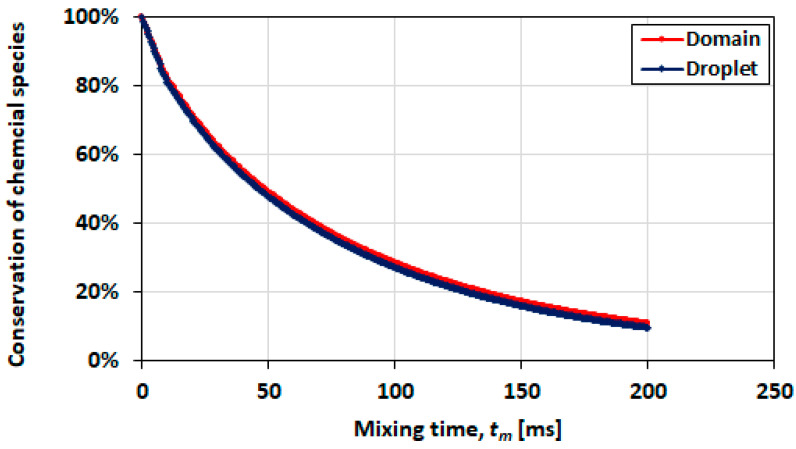
Conservation index for the diffusion-dominated mixing case =0.018, λ=2.

In contrast, the diffusion-dominated case (Figure 20) has unacceptably poor conservation of the chemical species which seem to disappear from both the droplet and the domain. In 50 ms, which is the order of diffusive mixing time, there is over 50% mass loss which extends to approximately 90% in 200 ms.

When the conservation indexes in the droplet of the two cases are contrasted for a mixing time of 6.5 ms, the losses for the diffusion-dominated case are just over 10%. This result demonstrates that the conservation index worsens with mixing time. Therefore, whilst the results for the diffusion-dominated cases may be in accurate due to these losses, the losses in the convection-dominated cases are negligibly small. When examining the case of diffusion only (i.e., the velocity field neglected), there is good conservation of the chemical species (approximately 100%) up to 5000 ms. The significance of this result is the demonstration that by itself, diffusion is not the cause of mass loss or leakage of the chemical species across the interface. The issue of mass loss emanates from the velocity field where diffusion dominates the mass transport physics. The results presented in the subsequent sections here are high confidence simulations where the convective mass transport dominates over diffusion and the two issues discussed in this sub-section (mass leakage and mass loss) are insignificant.

### 3.7. Effect of Initial Orientation of Solute Species

**Figure 21:**
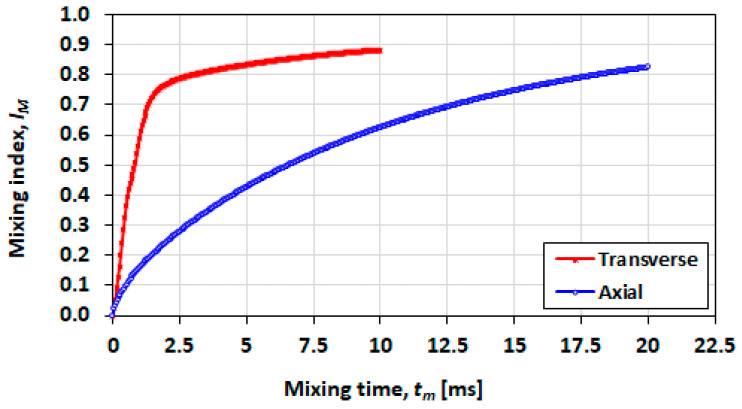
Effect of the initial mixing orientation on the mixing index for a droplet with initial length of $L_{0}=h$, Ca=0.21 and λ=0.21.

**Figure 22:**
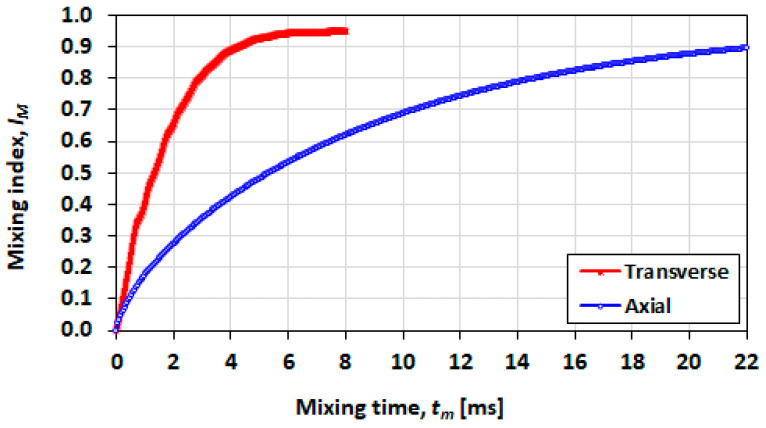
Effect of the initial mixing orientation on the mixing index for a droplet with initial length of $L_{0}=2h$, Ca=0.21 and λ=0.5.

**Figure 23:**
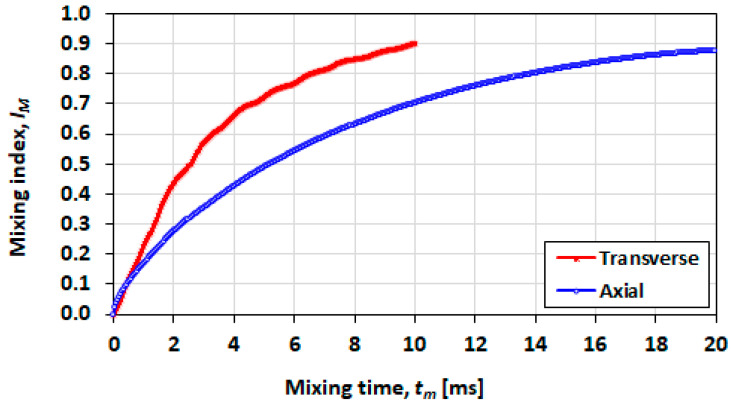
Effect of the initial mixing orientation on the mixing index for a droplet with initial length of $L_{0}=5h$, Ca=0.21 and λ=0.5.

In Figure 21, mixing performance is shown for a short droplet $(L_{0}=h$), in which the transverse initial orientation shows a distinct advantage in a shorter mixing time and a greater achievable final mixing index. In Figure 22 ($L_{0}=2h$) and Figure 23 ($L_{0}=5h$), it can be seen that the advantage decreases as the droplet length increases.

**Figure 24:**
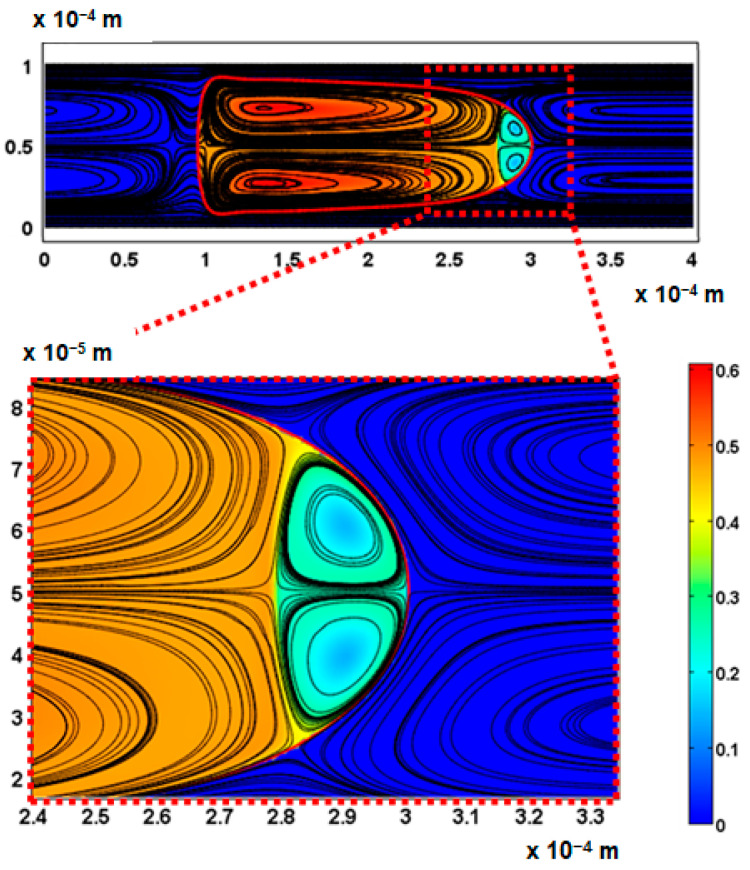
Influence of stagnation zones on poor mixing at the nose of the plug. The colour scale represents concentration (mol/m^3^).

The observation that the initial orientation of the solute has an influence of the efficiency of mixing supports previous experimental and numerical studies [9,36]. The nose region of the droplets exhibits poor mixing due to localised circulatory flow within the droplet as illustrated in Figure 24. At the centre of the recirculation, there is a stagnation zone where convective mixing is limited. The two stagnation zones on either side of the nose of the droplet/plug form a single region which ends up with a lower concentration than the average concentration in the rest of the plug.

In the cases discussed in following sections, only the transverse initial configuration is illustrated since it remains the more efficient option.

### 3.8. Effect of Droplet Size

**Figure 25:**
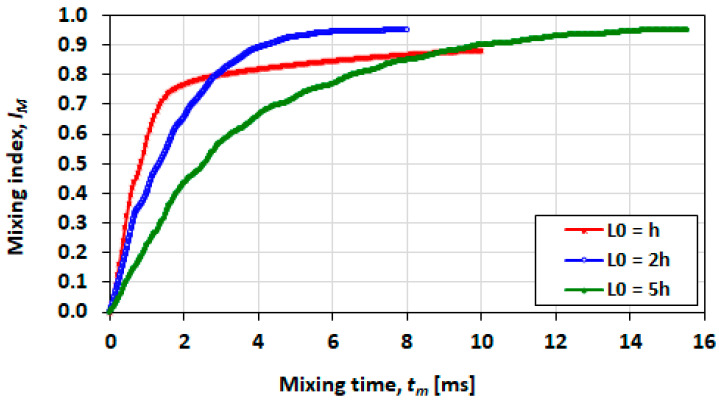
Comparison of mixing indexes for droplets with initial lengths of h, 2h and 5h at conditions of Ca=0.21  and λ=0.5.

Figure 25 shows plots of mixing indices versus mixing time for three droplet length cases ($L_{0}=h$, $L_{0}=2h$ and $L_{0}=5h$) obtained with mixing conditions of Ca=0.21 and λ=0.5. The initial orientation for the chemical species was transverse.

The smallest droplet ($L_{0}=h$) exhibits faster mixing in the first 2 ms, after which the rate of mixing drops reaches 0.9 in approximately 10 ms. In the case of the intermediate droplet ($L_{0}=2h$), the plateau is reached more slowly but the maximum index achieved is approximately 0.95. The longest droplet (plug) case has a slower rate of mixing and the mixing index of 0.95 is reached in 15 ms. Non-dimensional mixing distance $t_{m}U_{d}/L_{0}$ is of the same form.

The lower maximum mixing index reached in the case of the smallest droplet can be attributed to the large (20%) nose recirculation zone relative to the droplet size. In the other droplet cases of 2h and 5h, the recirculation zones accounts for 11% and 3%, respectively, where percentages refer to the area of the droplet. In the long plug, mixing is slower, and mixing is therefore optimal in the $L_{0}=2h$ case.

### 3.9. Effect of Capillary Number

**Figure 26:**
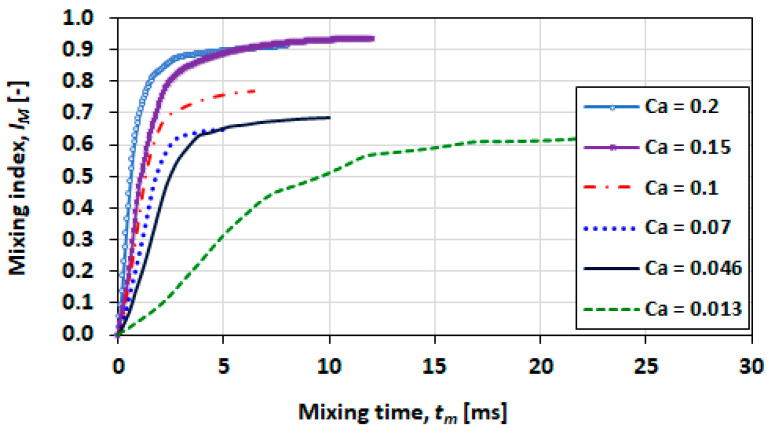
Mixing performance at various capillary numbers for λ=0.1.

**Figure 27:**
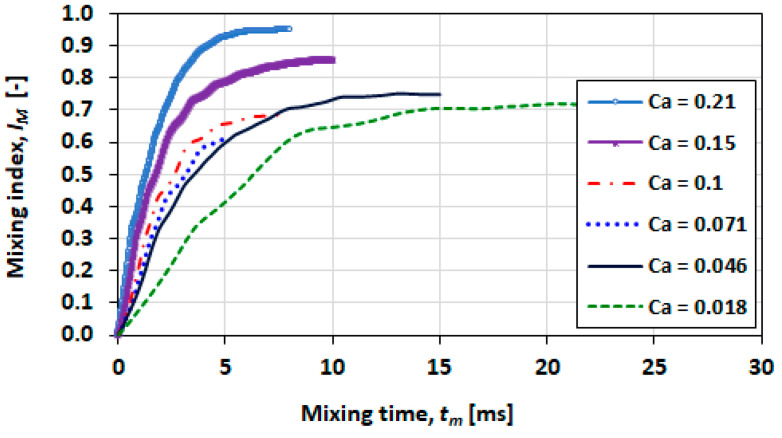
Mixing performance at various capillary numbers for λ=0.5.

The plots in Figure 26 and Figure 27 show the effect of the capillary number for viscosity ratios of 0.1 and 0.5, respectively. The capillary number in these cases is varied as a function of the superficial velocity. The investigation of these comparisons focussed only on the transverse orientation of the solute species.

The conditions of higher capillary numbers (>0.15) provide good mixing and mixing rate where $I_{M}$ reaches or exceeds 0.8 in 5 ms.

**Figure 28:**
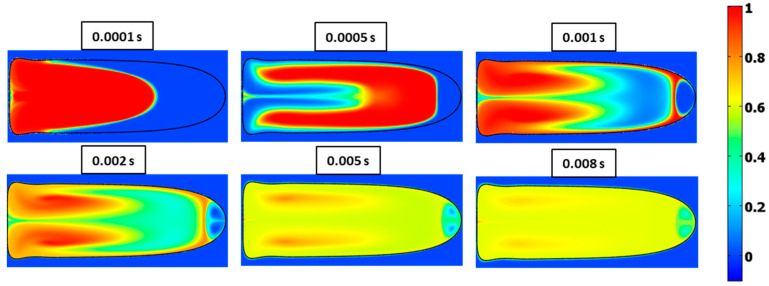
Surface plots of mixing at different times for Ca=0.21 and λ=0.5.

**Figure 29:**
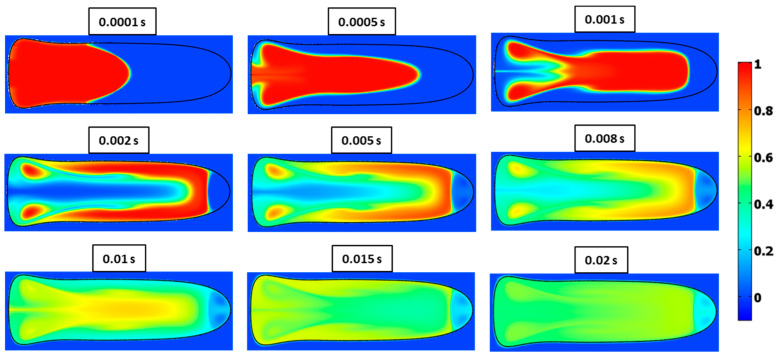
Surface plots of mixing at different times for Ca=0.21 and λ=2.

Contours of concentration at various times for Ca=0.21 and λ=0.5 are shown in Figure 28 and Figure 29 for Ca=0.21 and λ=2, respectively. The latter case exhibits slower mixing due to the higher viscosity of the fluid in the droplet.

### 3.10. Effect of Viscosity Ratio

**Figure 30:**
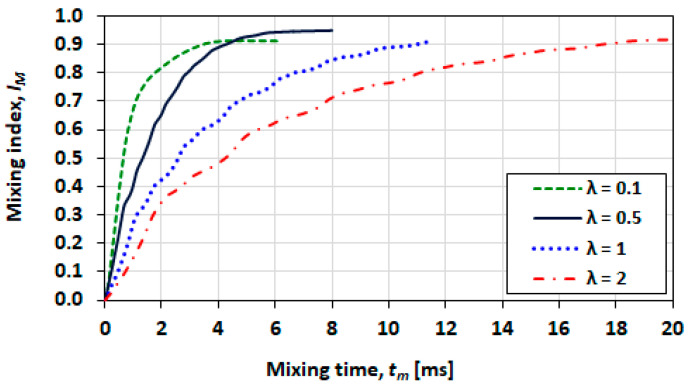
Comparison of mixing performances for various viscosity ratios at Ca=0.21.

The mixing index plots for various viscosity ratios at Ca=0.21 are given in Figure 30.

**Figure 31:**
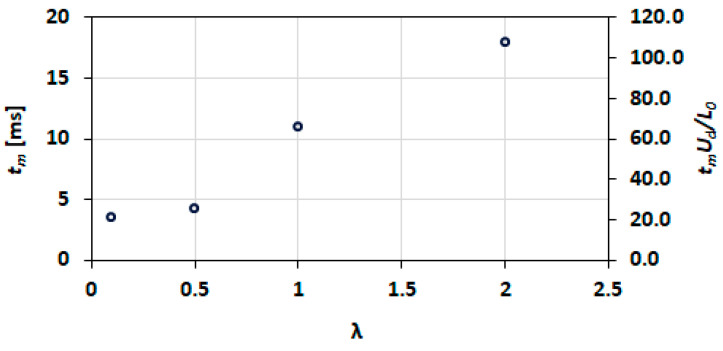
Influence of the viscosity ratio on mixing time and dimensionless mixing distance.

The final mixing index of the case for λ=0.5 is improved in comparison to that at λ=0.1. These results are counterintuitive, and no explanation is provided for this observation, at present. Figure 31 shows mixing time and dimensionless mixing distance as a function of viscosity ratio at Ca=0.21. The mixing time and the mixing distance both increase monotonically with the increasing viscosity ratio.

## 4. Discussion

Within the limitations of this study, the following observations were made: The droplet mobility increases with the capillary number, as expected, and decreases with the viscosity ratio λ as the droplet becomes less viscous and is more readily driven by shear at its boundary.

Flow fields showing the convection in the droplet reveal a significant recirculation zone in the front of the droplet. The length of this zone decreases significantly with Ca for λ=0.5, but is not noticeably changed for λ=2.

As expected, transverse initial orientation of the species in the droplet promotes faster mixing, but mixing performance is impeded by the recirculation cap. Mixing in short droplets is initially faster than in longer slugs but reaches a lower maximum value; longer droplets achieve better final mixing, but over a longer time for the same Ca and λ.

Overall, Ca=0.2 and Ca=0.15 provide the best mixing performance for cases where λ<1. The latter cases are convection dominated and are able to achieve mixing indices of above 0.85. There are three types of mixing behaviour: firstly, convection-dominated mixing where at least $I_{M}$ of 0.8 is achieved within 5 ms; secondly, convention mixing within 5 ms, after which diffusion becomes dominant and the rate of mixing then plateaus, and the values of $I_{M}$ do not reach 0.8; and thirdly, a diffusion-dominated mixing where the mixing rate is low and it takes at least 15 ms for the rate of mixing to change before it begins to plateau.

At the flow conditions studied, a maximum mixing index of one cannot practically be achieved due to the recirculation at the droplet nose. In the case where the carrier fluid has a lower viscosity than the droplet (λ=0.5), the mixing is demonstrably better than the contrasting case of viscosity ratio of 2. At *Ca* =0.2, a mixing index of 0.8 is achieved in 5 ms at λ=2. The same mixing index is achieved in double the time at λ=0.5. Higher capillary numbers exhibit better mixing but also require long microchannels as the droplets travel at high speeds. At capillary numbers of 0.1 and below, the mixing index curve reaches the asymptotic value of approximately 0.8 or below. This is due to the influence of the stagnation zones. The viscosity ratio of the droplet phase and the carrier phase does influence the flow field and, therefore, the quality of mixing in the droplet. Under conditions of similar or comparable capillary numbers, better mixing is obtained when the viscosity of the carrier fluid is lower than the droplet phase.

## 5. Conclusions

This work has demonstrated the use of the moving frame of reference approach and the two-phase level set method in a finite element solver for studying mixing in a droplet or plug moving in a straight microchannel.

The key conclusions of this work are that (1) a limitation of the method exists for flow conditions where the droplet mobility approaches unity, due to the moving wall boundary condition, which results in an untenable solution under those conditions; (2) the efficiency of the mixing declines as the length of the droplet or plug increases; (3) the initial orientation of the droplet influences the mixing and the transverse orientation provides better mixing performance than the axial orientation and; (4) the recirculation inside the droplet depends on the superficial velocity and the viscosity ratio.

This work has demonstrated that both the viscosities of the individual phases, and their viscosity ratios influence the extent and speed of mixing and the maximum mixing index that can be reached. In the cases illustrated here, the development of a recirculation zone in the leading region of the droplet, and its dependence on droplet length and viscosity ratio, has been shown.

In previous work, the contribution of the viscosity in the droplet phase has a tendency to be neglected, and the assumption made that the capillary number on its own can be used to characterise the droplet flow. This work provides the first approximation approach, and an extension to three dimensions would be useful in considering the flow field in the recirculating droplet cap and the influence of confinement.

## Data Availability

The data presented in this study are available on request from the corresponding author.
